# Use of Cigarettes, Cannabis, and Alcohol Among Asian American, Native Hawaiian, and Pacific Islander Adults: Community-Based National Survey Analysis

**DOI:** 10.2196/76465

**Published:** 2025-08-20

**Authors:** Vuong Van Do, Van My Ta Park, Nhung Nguyen, Pamela May Ling, Marian Tzuang, Bora Nam, Marcelle M Dougan, Oanh L Meyer, Janice Y Tsoh

**Affiliations:** 1 Center for Tobacco Control Research and Education, University of California San Francisco, San Francisco, CA, United States; 2 Asian American Research Center on Health, University of California San Francisco, San Francisco, CA, United States; 3 Department of Community Health Systems, School of Nursing, University of California San Francisco, San Francisco, CA, United States; 4 Division of General Internal Medicine, Department of Medicine, University of California San Francisco, San Francisco, CA, United States; 5 Department of Public Health and Recreation, San José State University, San Jose, United States; 6 Department of Neurology, University of California, Davis, Davis, United States; 7 Department of Psychiatry and Behavioral Sciences, School of Medicine, University of California San Francisco, 675 18th Street, Nancy Friend Pritzker Psychiatry Building, San Francisco, CA, United States

**Keywords:** cigarette smoking, cannabis, alcohol, Asian Americans, Native Hawaiians and Pacific Islanders

## Abstract

**Background:**

Asian American, Native Hawaiian, and Pacific Islander (AANHPI) populations have diverse cultural, immigration, and sociodemographic characteristics. Aggregated data could mask substantial differences in substance use between cultural subgroups in this population. Yet, studies examining substance use among the AANHPI population are limited.

**Objective:**

This study aimed to describe cigarette, cannabis, and alcohol use among AANHPI adults by cultural subgroup and sex.

**Methods:**

We analyzed data from 3411 AANHPI respondents of a multilingual national survey “COMPASS” during December 2021-May 2022. Primary outcomes were self-report current (every day or some days) use of cigarettes, cannabis, and alcohol. Cultural subgroups included Asian Indian, Ethnic Chinese, Filipino, Japanese, Korean, Native Hawaiian and Pacific Islander, Vietnamese, other cultural groups, and multicultural groups. Other covariates include sex, other sociodemographics, experiences of discrimination (Everyday Discrimination Scale), and mental health (Patient Health Questionnaire 4). Multivariable logistic regressions were used to examine correlates of each substance use among AANHPI adults.

**Results:**

The prevalence of current cigarette, cannabis, and alcohol use was 4.2% (142/3359), 5.5% (184/3235) and 37.6% (1265/3361), respectively. Cigarette use ranged from 1.0% (1/100) in Asian Indian females to 14.8% (10/71) in multicultural males. Cannabis use ranged from 1.9% among Asian Indian (1/53) and Vietnamese males (4/211) to 15.7% (11/70) in multicultural females. Alcohol use varied from 6.6% (4/61) in Native Hawaiian and Pacific Islander females to 56.3% (40/71) among multicultural males. Male participants with elevated depression and anxiety symptoms were more likely to report using all 3 substances than males with minimal symptoms. However, depression and anxiety symptoms were only associated with cannabis and alcohol use among female participants. US-born female participants were more likely to report using all 3 substances compared to foreign-born females, while being US-born was only associated with higher odds of alcohol use among males. Perceived discriminatory experience was associated with higher odds of smoking in both sexes and alcohol drinking in males.

**Conclusions:**

Cigarette smoking, cannabis, and alcohol use varied widely across AANHPI cultural groups, between and within each sex. These findings underscore the necessity to disaggregate data for substance use behaviors to guide health policy and intervention programs for AANHPI adults.

## Introduction

Tobacco, cannabis, and alcohol are the most commonly used substances among US adults, and tobacco and alcohol are leading causes of morbidity and mortality, especially cancer [1]. Among the Asian American, Native Hawaiian, and Pacific Islander (AANHPI) population, cancer has been the leading cause of death since 2000, with lung and liver cancer deaths the two most common among men and ranked first and fourth among women [2,3]. In addition, the 5‐year cancer‐specific survival for AANHPI men (62%) was lower compared to non‐Latinx White American men (68%) [2]. In addition, people who misuse one substance are also more likely to use other illicit drugs [4,5], including methamphetamine and fentanyl, the two leading causes of drug-related deaths among Asian Americans and Native Hawaiians and other Pacific Islanders (NHPI) [6]. One of the proposed strategies to reduce cancer and drug overdose mortality among the AANHPI population is to improve cancer screening and implement behavioral interventions to reduce substance use among high-risk groups [2]. Yet, studies examining substance use among the AANHPI population are limited.

According to data from the 2022 National Survey on Drug Use and Health, past month substance use prevalence among English-speaking US adults was 15.9% for cigarettes and cannabis and 52.9% for alcohol [7]. AANHPI adults are often combined into one or two groups in national reports on substance use to compare with other race and ethnic groups, which shows a lower prevalence of substance use among this group compared with other racial or ethnic groups [8,9]. In addition, the stereotype that AANHPI groups are model minority populations has negatively impacted data equity [10,11], leading to an underrepresentation of the AANHPI subgroups in clinical research [12]. However, the AANHPI population has many cultural subgroups with diverse cultural, immigration, and sociodemographic characteristics. Therefore, aggregated data could mask substantial differences in substance use between cultural subgroups in this population.

About 20.6 million people self-identify as AANHPI (not in combination with another race), accounting for about 6.2% of the total US population [13]. The Department of Health and Human Services provides guidelines for collecting standard racial and ethnic information such that the Asian American category is divided into seven subgroups (Asian Indian, Chinese, Filipino, Japanese, Korean, Vietnamese, and other Asians), and the NHPI group categorized into four subgroups (Native Hawaiian, Guamanian or Chamorro, Samoan, and Other Pacific Islanders) [14]. Despite these guidelines, AANHPI subgroups are often under-sampled in national surveys [15], preventing the disaggregation of data among this population. A few studies have examined tobacco use among the AANHPI population and found a wide range in the prevalence of use, including two national surveys [16,17], both of which were English-administered surveys and collected data more than a decade ago. In addition, a community-based survey of 306 NHPI adults during the COVID-19 pandemic found a high prevalence of smoking, alcohol, and substance use among this population [18]. Significant sex differences in substance use have also been noted among AANHPI populations, with males generally at higher risk of substance use compared to females [16,17,19]. Thus, it is crucial to monitor tobacco and other substance use within each sex across AANHPI subgroups, including both English and non-English speakers.

Disaggregated data are instrumental to understanding subgroup differences and addressing substance-use disparities that might otherwise go unnoticed. This effort can inform effective health policies and behavior change interventions to address the unique challenges faced by subgroups with a higher rate of substance use within AANHPI communities. To fill the literature gap, this study examined cigarettes, cannabis, and alcohol use among AANHPI adults, disaggregated by cultural subgroups and sexes using data from a national survey administered in English and multiple Asian languages.

## Methods

### Data Sources and Participants

This study used data from the “COVID-19 Effects on the Mental and Physical Health of Asian Americans & Pacific Islanders Survey Study” (COMPASS), a community-based national survey that assesses COVID-19 effects on AANHPI adults. Data collection of the first survey (COMPASS I) occurred during October 2020 and January 2021, with 5420 participants completing the survey. Details about participant recruitment and study procedures have been reported elsewhere [20]. Briefly, eligibility criteria included self-identification as AANHPI alone or in combination with other races and ethnicities, being able to read English or Chinese (traditional and simplified Chinese) or Korean or Samoan or Vietnamese (the survey languages), being 18 years old or older, and residing in the United States. Participants were recruited through community partners who served AANHPI communities, personal or professional networks, social media, ethnic media, and through the Collaborative Approach for Asian Americans, Native Hawaiians, and Pacific Islanders Research and Education registry [21]. Participants completed the survey by themselves via the study website or were interviewed by research staff over the phone or in person. Data were collected using REDCap (Research Electronic Data Capture; Vanderbilt University) [22].

COMPASS I participants were invited to complete a follow-up survey (COMPASS II) between December 2021 and May 2022. This study analyzed data from the 3411 participants who completed the COMPASS II survey.

### Measures

#### Cigarette, Cannabis, and Alcohol Use

Participants were asked, “Do you now smoke cigarettes?” Similar questions were asked for cannabis and alcohol use. Response options were “every day,” “some days,” and “not at all.” “Every day” or “some days” responses were coded as current use of the substance.

#### Cultural Subgroups

Cultural subgroups were self-reported and classified into Asian Indian, Ethnic Chinese, Filipino, Japanese, Korean, NHPI, Vietnamese, Other Asian group, and multicultural groups. The Ethnic Chinese group included mainland Chinese, Hongkongers, Taiwanese, and Huaren. The multicultural group included participants who self-identified with two or more cultural groups.

#### Other Covariates

Perceived discriminatory experience (yes or no) was measured using a revised 8-item everyday discrimination scale [23]. Experience of discrimination was coded “yes” if participants reported any of the 8 items in the past 6 months. Depression and anxiety symptoms were measured using the Patient Health Questionnaire 4 (PHQ-4) and categorized as normal, mild, and moderate or severe levels [24]. Self-reported English proficiency was coded as limited if participants reported any of their abilities for speaking, reading, or writing English as “some,” “a little,” or “not at all.” Other demographic covariates included age, biological sex (female vs male), education, marital status, census region, and nativity (US-born vs foreign-born).

### Ethical Considerations

The institutional review board at the University of California San Francisco reviewed and approved the COMPASS study (UCSF IRB: #20‐31925). Participants were provided with detailed information about the study, and informed consent was obtained before survey completion. Participants received a US $10 gift card upon survey completion. Personal details of participants were kept confidential, and a unique study identification number was assigned to each participant for data management. Deidentified data were used for the analysis.

### Statistical Analysis

Data were analyzed from April 15 to May 30, 2024, using Stata version 17 (StataCorp LLC). Descriptive statistics were used to describe the characteristics of participants and substance use. Multivariable logistic regression models were used to examine the association between each type of substance use and the cultural group and other covariates for each sex. To compare the odds of using a substance between a cultural subgroup and the rest of the AANHPI adults, we repeated regression models for the dummy variable of each cultural subgroup. All statistical tests were 2-sided, and *P*<.05 was considered statistically significant.

## Results

### Participant Characteristics

Participant characteristics are provided in Table 1. Among 3411 participants, the majority self-identified as ethnic Chinese (n=1176, 34.5%), Korean (n=692, 20.3%), and Vietnamese (n=669, 19.6%). Participants from Japanese, Asian Indian, Filipino, and NHPI culture groups each accounted for less than 5% of the sample. Most participants were female (n=2262, 66.3%), had a bachelor’s degree or higher (n=2527, 74.1%), were born abroad (n=2175, 63.8%), and reported recent discriminatory experiences (n=2050, 60.1%).

**Table 1.** Characteristics of participants in the COMPASS II study in 2021 (n=3411).

Characteristics	Values n (%)
Cultural group	
Asian Indian	156 (4.6)
Ethnic Chinese^a^	1176 (34.5)
Filipino	115 (3.4)
Japanese	165 (4.8)
Korean	692 (20.3)
Native Hawaiians and Pacific Islanders	95 (2.8)
Vietnamese	669 (19.6)
Other cultural group	118 (3.5)
Multicultural group	225 (6.6)
Age group (years)	
18‐29	695 (20.4)
30‐49	1250 (36.6)
50‐64	979 (28.7)
65+	487 (14.3)
Sex	
Male	1149 (33.7)
Female	2262 (66.3)
Education	
Less than high school	160 (4.7)
Completed high school or equivalent	344 (10.1)
Some college no degree	343 (10.1)
Bachelor’s degree or higher	2527 (74.1)
Missing	37 (1.1)
Marital status	
Single	901 (26.4)
Married or living with a partner	2240 (65.7)
Separated, divorced, or widowed	250 (7.3)
Missing	20 (0.6)
Census Region	
Midwest	289 (8.5)
Northeast	344 (10.1)
South	472 (13.8)
West	2303 (67.6)
Nativity	
US-born	1236 (36.2)
Foreign born	2175 (63.8)
Limited English proficiency	
Yes^b^	657 (19.3)
No	2754 (80.7)
Perceived discriminatory experience	
Yes	2050 (60.1)
No	1320 (38.7)
Missing	41 (1.2)
Depression and anxiety symptoms^c^	
Normal	2215 (64.9)
Mild	853 (25.0)
Moderate or severe	343 (10.1)

a Ethnic Chinese includes mainland Chinese, Hongkonger, Taiwanese, and Huaren.

b Self-reported English proficiency was categorized as limited (“yes”) if speaking or reading or writing English indicated as “some,” “a little,” or “not at all.”

c Depression and anxiety symptoms were measured using 4 items from the Patient Health Questionnaire 4 (PHQ-4).

### Prevalence of Cigarette, Cannabis, and Alcohol Use by Cultural Subgroups and Sex

The proportions of current substance use among AANHPI adults by cultural groups and sex are presented in Table 2. The overall prevalence of cigarette smoking among AANHPI adults was 4.2% (142/3359), with 8.4% (94/1123) of males and 2.1% (48/2236) of females reporting current smoking. Among male AANHPI adults, the prevalence of current cigarette smoking varied widely from 3.7% (2/54) among Asian Indians to 9.8% (21/214) among Vietnamese and 14.8% (10/71) among the multicultural group. The prevalence of current cigarette smoking among AANHPI females was low (<3%) in most cultural subgroups, except for NHPI (4/61, 6.6%) and those from the multicultural group (8/153, 5.2%).

**Table 2.** Cigarette smoking, cannabis, and alcohol use among Asian Americans, Native Hawaiians, and Pacific Islanders (AANHPI) by cultural subgroups and sex.

		Current use (every day or some days)		
Cultural subgroups		Cigarette (n=3359), n/N (%)	Cannabis (n=3325), n/N (%)	Alcohol (n=3361), n/N (%)
Asian Indian				
	Overall	3/154 (1.9)	5/153 (3.3)	58/153 (37.9)
	Male	2/54 (3.7)	1/53 (1.9)	29/53 (54.7)
	Female	1/100 (1.0)	4/100 (4.0)	29/100 (29.0)
Ethnic Chinese				
	Overall	34/1157 (2.9)	63/1146 (5.5)	415/1159 (35.8)
	Male	23/379 (6.1)	25/375 (6.7)	156/382 (40.8)
	Female	11/778 (1.4)	38/771 (4.9)	259/777 (33.3)
Filipino				
	Overall	5/113 (4.4)	12/113 (10.6)	68/114 (59.7)
	Male^a^	3/31 (9.7)^a^	3/31 (9.7)^a^	24/32 (75.0)^a^
	Female	2/82 (2.4)	9/82 (11.0)	44/82 (53.7)
Japanese				
	Overall	8/161 (5.0)	9/158 (5.7)	71/163 (43.6)
	Male^a^	5/45 (11.1)^a^	3/45 (6.7)^a^	23/47 (48.9)^a^
	Female	3/116 (2.6)	6/113 (5.3)	48/116 (41.4)
Korean				
	Overall	33/676 (4.9)	22/670 (3.3)	316/679 (46.5)
	Male	23/266 (8.6)	8/263 (3.0)	141/265 (53.2)
	Female	10/410 (2.4)	14/407 (3.4)	175/414 (42.3)
NHPI^b^				
	Overall	9/95 (9.5)	5/94 (5.3)	7/95 (7.4)
	Male^a^	5/34 (14.7)^a^	2/34 (5.9)^a^	3/34 (8.8)^a^
	Female	4/61 (6.6)	3/60 (5.0)	4/61 (6.6)
Vietnamese				
	Overall	28/661 (4.2)	29/653 (4.4)	167/658 (25.4)
	Male	21/214 (9.8)	4/211 (1.9)	69/213 (32.4)
	Female	7/447 (1.6)	25/442 (5.7)	98/445 (22.0)
Other cultural group				
	Overall	4/118 (3.4)	10/117 (8.5)	59/118 (50.0)
	Male^a^	2/29 (6.9)^a^	4/29 (13.8)^a^	20/29 (69.0)^a^
	Female	2/89 (2.2)	6/88 (6.8)	39/89 (43.8)
Multicultural group				
	Overall	18/224 (8.0)	29/221 (13.1)	104/222 (46.8)
	Male	10/71 (14.8)	11/70 (15.7)	40/71 (56.3)
	Female	8/153 (5.2)	18/151 (11.9)	64/151 (42.4)
Overall AANHPI		142/3359 (4.2)	184/3235 (5.5)	1265/3361 (37.6)
Male AANHPI		94/1123 (8.4)	61/1111 (5.5)	505/1126 (44.8)
Female AANHPI		48/2236 (2.1)	123/2214 (5.6)	760/2253 (34.0)

a Unstable estimates (sample size <50)

b NHPI: Native Hawaiians and Pacific Islanders.

The overall prevalence of cannabis use was 5.5% (184/3235) among AANHPI adults, with the prevalence almost equal between males and females. Cannabis use prevalence was highest among Filipinos (12/113, 10.6%) and multicultural participants (29/221, 13.1%). Among males, cannabis use ranged widely from 1.9% in participants of Asian Indian (1/53) and Vietnamese (4/211) cultures to 15.7% (11/70) in those of the multicultural group. Cannabis use was highest among females of the multicultural group (18/151, 11.9%) and Filipino (9/82, 11%). In addition, cannabis use was more prevalent among females than males for participants of Vietnamese (5.7% [25/442] vs 1.9% [4/211]) and Asian Indian (4% [4/100] vs 1.9% [1/53]) cultures.

Alcohol use was common among AANHPI adults (1265/3361, 37.6%) and more common among males (505/1126, 44.8%) than females (760/2235, 34%). Among cultural subgroups, the prevalence of current alcohol use varied from 7.4% (7/95) among NHPIs to 59.7% (68/114) among Filipinos. Male participants of Asian Indian (58/153, 54.7%), Korean (141/265, 53.2%), or a multicultural group (40/71, 56.3%) were most likely to report current alcohol use. Among females, alcohol use was most common among Filipinos (44/82, 53.7%), Koreans (175/414, 42.3%), Japanese (48/116, 41.4%), and the multicultural group (64/151, 42.4%).

### Correlates of Substance Use Among AANHPI Males

Among male participants, there were no statistically significant differences in the odds of cigarette smoking between each cultural subgroup compared with the rest of the AANHPI adults (Table 3). However, compared with the rest of the AANHPI males, Vietnamese males were less likely (adjusted odds ratio [AOR] 0.26, 95% CI 0.09‐0.77) to report cannabis use, whereas those males of the multicultural group had higher odds of reporting cannabis use (AOR 2.39, 95% CI 1.10‐5.19). In addition, Korean males had higher odds of alcohol use (AOR 1.41, 95% CI 1.02‐1.95) compared with other males, while Ethnic Chinese males were less likely to report alcohol use (AOR 0.63, 95% CI 0.48‐0.83) compared with their other male peers.

**Table 3.** Associated factors of cigarette smoking, cannabis, and alcohol use among Asian Americans and Native Hawaiians and Pacific Islander males.

	Current use (daily or some days), AOR^a^ (95% CI)		
Characteristics	Cigarette (n=1096)	Cannabis (n=1082)	Alcohol (n=1097)
Cultural group^b^			
Asian Indian	0.55 (0.12‐2.44)	0.47 (0.06‐3.73)	1.72 (0.95‐3.12)
Ethnic Chinese	0.66 (0.39‐1.11)	1.23 (0.69‐2.18)	0.63^c^ (0.48‐0.83)
Filipino	—^d^	—	—
Japanese	—	—	—
Korean	1.15 (0.65‐2.05)	0.59 (0.26‐1.36)	1.41^e^ (1.02‐1.95)
NHPI^f^	—	—	—
Vietnamese	1.32 (0.73‐2.39)	0.26^e^ (0.09‐0.77)	0.80 (0.57‐1.14)
Other cultural group	—	—	—
Multicultural group	1.80 (0.83‐3.90)	2.39^e^ (1.10‐5.19)	1.58 (0.95‐2.63)
Age group (years)			
18‐29	Reference	Reference	Reference
30‐49	1.19 (0.52‐2.69)	0.32^c^ (0.15‐0.70)	1.58^e^ (1.02‐2.44)
50‐64	0.87 (0.34‐2.21)	0.19^c^ (0.07‐0.52)	1.00 (0.62‐1.62)
65+	0.36 (0.12‐1.13)	0.10^c^ (0.02‐0.45)	0.75 (0.43‐1.31)
Education			
Less than high school	Reference	Reference	Reference
Completed high school or equivalent	0.40^e^ (0.16‐0.99)	0.18^e^ (0.03‐0.88)	0.53 (0.25‐1.15)
Some college no degree	0.32^e^ (0.11‐0.99)	0.10^c^ (0.02‐0.56)	0.77 (0.34‐1.75)
Bachelor’s degree or higher	0.16^g^ (0.06‐0.41)	0.13^c^ (0.03‐0.58)	0.72 (0.35‐1.49)
Limited English proficiency	1.61 (0.81‐3.21)	0.69 (0.19‐2.59)	0.97 (0.63‐1.49)
Marital status			
Single	Reference	Reference	Reference
Married or living with a partner	1.59 (0.79‐3.19)	0.96 (0.47‐1.95)	1.17 (0.81‐1.67)
Separated, divorced, or widowed	6.19^g^ (1.97‐19.53)	0.74 (0.08‐7.07)	1.30 (0.58‐2.93)
US-born vs foreign born	1.14 (0.63‐2.09)	1.39 (0.73‐2.66)	1.53^e^ (1.11‐2.11)
Perceived discriminatory experience	2.08^i^ (1.19‐3.61)	1.42 (0.74‐2.74)	1.31^e^ (1.01‐1.72)
Depression and anxiety symptoms^h^			
Normal	Reference	Reference	Reference
Mild	2.11^c^ (1.25‐3.55)	1.91 (0.97‐3.76)	1.40^e^ (1.02‐2.44)
Moderate or severe	2.80^c^ (1.42‐5.51)	5.41^g^ (2.58‐11.37)	1.00 (0.64‐1.59)
Census region			
Midwest	Reference	Reference	Reference
Northeast	1.81 (0.60‐5.44)	1.20 (0.27‐5.22)	0.98 (0.55‐1.75)
South	1.97 (0.71‐5.49)	1.92 (0.53‐6.97)	0.67 (0.41‐1.17)
West	1.24 (0.47‐3.31)	2.45 (0.75‐8.03)	0.80 (0.50‐1.29)

a AOR: adjusted odds ratio.

b AOR for cultural subgroups were from comparisons between a specific subgroup and all the rest of the participants, using different regressions that controlled for the same covariates.

c *P*<.01.

d Not available.

e *P*<.05 .

f NHPI: Native Hawaiians and Pacific Islanders.

g *P*<.001.

h Depression and anxiety symptoms were measured using 4 items from the Patient Health Questionnaire 4 (PHQ-4). Estimates were not produced for Filipino, Japanese, NHPI, and other Asian American groups due to the small sample size of these groups.

The perceived discriminatory experience was significantly associated with higher odds of smoking (AOR 2.08, 95% CI 1.19‐3.61) and drinking alcohol (AOR 1.31, 95% CI 1.01‐1.72) but not using cannabis. Male participants with moderate or severe depression and anxiety symptoms were almost 3 times more likely to report smoking (95% CI 1.42‐5.51) and 5 times more likely to report cannabis use (95% CI 2.58‐11.37) compared with those who had a normal level of depression and anxiety symptoms. Being born in the United States was associated with higher odds of drinking alcohol, but not for cigarette and cannabis use. English proficiency was not found to be associated with using any of these 3 substances. In addition, male participants of older age groups were less likely to report cannabis use than young adults aged 18‐29 years, and those with higher levels of education were also less likely to report smoking and cannabis use compared to those with less than high school education.

### Correlates of Substance Use Among AANHPI Females

Factors associated with substance use among female participants are shown in Table 4. The odds of smoking among female Koreans were twice (AOR 2.27, 95% CI 1.04‐4.98) that of other non-Korean AANHPI females. Female participants of the multicultural group were about 2 times more likely to report cannabis use (AOR 1.89, 95% CI 1.06‐3.37) compared with the rest. Female participants with Filipino (AOR 1.84, 95% CI 1.15‐2.93) and Korean (AOR 1.70, 95% CI 1.32‐2.19) cultural backgrounds were more likely to report alcohol use, while those females with ethnic Chinese, Vietnamese, and NHPI backgrounds were less likely to report alcohol use compared with females of the rest.

**Table 4.** Associated factors of cigarette smoking, cannabis, and alcohol use among Asian Americans and Native Hawaiians and Pacific Islander females.

	Current use (daily or some days), AOR^a^ (95% CI)		
Characteristics	Cigarette (n=2173)	Cannabis (n=2151)	Alcohol (n=2174)
Cultural group^b^			
Asian Indian	0.61 (0.08‐4.77)	0.86 (0.30‐2.51)	0.70 (0.44‐1.13)
Ethnic Chinese	0.50 (0.25‐1.04)	0.66 (0.43‐1.01)	0.78^c^ (0.64‐0.95)
Filipino	1.16 (0.26‐5.30)	1.46 (0.67‐3.18)	1.84^c^ (1.15‐2.93)
Japanese	0.74 (0.16‐3.32)	0.99 (0.40‐2.46)	1.40 (0.92‐2.12)
Korean	2.27^c^ (1.04‐4.98)	0.84 (0.46‐1.55)	1.70^d^ (1.32‐2.19)
Native Hawaiians and Pacific Islanders	2.49 (0.72‐8.63)	1.06 (0.21‐5.30)	0.27^c^ (0.08‐0.90)
Vietnamese	0.63 (0.26‐1.53)	1.09 (0.66‐1.82)	0.64^e^ (0.48‐0.84)
Other cultural group	0.74 (0.14‐3.93)	1.60 (0.57‐4.49)	1.10 (0.67‐1.80)
Multicultural group	2.11 (0.91‐4.91)	1.89^c^ (1.06‐3.37)	1.29 (0.90‐1.83)
Age group (years)			
18‐29	Reference	Reference	Reference
30‐49	4.46^e^ (1.71‐11.62)	0.35^d^ (0.20‐0.63)	0.70^c^ (0.52‐0.94)
50‐64	3.66^c^ (1.22‐10.94)	0.35 (0.93‐10.54)	0.56^e^ (0.40‐0.79)
65+	2.72 (0.65‐5.84)	0.06^e^ (0.01‐0.31)	0.45^d^ (0.29‐0.69)
Education			
Less than high school	Reference	Reference	Reference
Completed high school or equivalent	0.22^c^ (0.06‐0.85)	0.12^e^ (0.02‐0.58)	0.63 (0.28‐1.40)
Some college no degree	0.33 (0.09‐1.20)	0.45 (0.11‐1.80)	1.31 (0.62‐2.75)
Bachelor’s degree or higher	0.07^d^ (0.02‐0.26)	0.24^c^ (0.06‐0.93)	1.86 (0.92‐3.75)
Limited English proficiency	0.75 (0.25‐2.26)	0.34 (0.10‐1.12)	0.53^d^ (0.37‐0.75)
Marital status			
Single	Reference	Reference	Reference
Married or living with a partner	0.34^e^ (0.16‐0.72)	1.10 (0.66‐1.84)	1.10 (0.84‐1.43)
Separated, divorced, or widowed	0.36 (0.10‐1.21)	2.61^c^ (1.07‐6.34)	1.19 (0.76‐1.85)
US-born vs Foreign born	3.73^c^ (1.68‐8.30)	2.73^d^ (1.70‐4.38)	1.42^e^ (1.14‐1.77)
Perceived discriminatory experience	2.21^c^ (1.02‐4.80)	1.25 (0.80‐1.94)	1.00 (0.82‐1.23)
Depression and anxiety symptoms^f^			
Normal	Reference	Reference	Reference
Mild	1.35 (0.67‐2.73)	1.72^c^ (1.09‐2.72)	1.35^e^ (1.08‐1.68)
Moderate or severe	1.78 (0.76‐4.13)	2.63^d^ (1.56‐4.44)	1.46^c^ (1.07‐1.98)
Census region			
Midwest	Reference	Reference	Reference
Northeast	1.59 (0.31‐8.11)	6.02^e^ (1.81‐20.02)	1.00 (0.64‐1.57)
South	2.20 (0.48‐10.04)	3.14 (0.93‐10.54)	1.02 (0.67‐1.56)
West	1.47 (0.37‐5.84)	3.91^c^ (1.29‐11.83)	0.75 (0.52‐1.08)

a AOR: adjusted odds ratio.

b AORs for cultural subgroups were from comparisons between a specific subgroup and all the rest of the participants, using different regressions that controlled for the same covariates.

c *P*<.05.

d *P*<.001

e *P*<.01.

f Depression and anxiety symptoms were measured using 4 items from the Patient Health Questionnaire 4 (PHQ-4).

The perceived discriminatory experience was significantly associated with smoking (AOR 2.21, 95% CI 1.02‐4.80) among female participants but not for other substances, whereas depression and anxiety symptoms were not associated with smoking. However, female participants with elevated depression and anxiety symptoms were more likely to report using cannabis and alcohol than those females with a normal level of depression and anxiety symptoms. Being born in the United States was significantly associated with a higher likelihood of using all 3 substances among AANHPI females. Older females were more likely to smoke cigarettes than young females between ages 18 and 29 years, but the relationship was the opposite for cannabis and alcohol use. Limited English proficiency was significantly associated with lower odds of alcohol drinking (AOR 0.53, 95% CI 0.37‐0.75) but not cigarette and cannabis use. In addition, females of older age groups were more likely to report smoking but less likely to report cannabis and alcohol use compared to their young adult counterparts aged 18‐29 years. Female participants with higher levels of education were also less likely to report smoking and cannabis use compared to those with less than high school education.

## Discussion

### Principal Findings

Given that disaggregated data about substance use by sex among the AANHPI communities are scarce, this study adds several important findings to the literature. First, our study showed different patterns of use of 3 substances, including cigarettes, cannabis, and alcohol, among a nationally recruited sample of AANHPI adults. Second, the study provided disaggregated data for each type of substance use and indicated a wide variation in the prevalence of use by cultural subgroups within and between each sex. For example, smoking was mostly among AANHPI males and ranged from 3.7% among Asian Indians to 14.8% among the multicultural group; whereas, the rates of cannabis use among AANHPI adults were similar between the 2 sexes, and Filipinos reported the highest rate of current cannabis use. Alcohol use was common among males of some subgroups, including Filipino, Indian, and Korean. Third, findings revealed different factors associated with each type of substance use within each sex.

Despite a low overall prevalence of cigarette smoking (4.2%) among AANHPI adults, the prevalence among AANHPI males was almost four times higher than among AANHPI females. The sex disparity in smoking remained consistent with findings from previous studies [16,17]. In addition, smoking prevalence varied widely across cultural subgroups among male AANHPI adults. Particularly, participants of Vietnamese (9.8%) and multicultural subgroups (14.8%) had the highest prevalence of current smoking, which was comparable to estimates for general US adults in 2022 (11.6%) or adults of other racial/ethnic groups such as non-Hispanic White (12.7%), non-Hispanic Black (14.2%), and Hispanic (8.0%) [25] of the National Health Interview Survey. Our finding confirms the need for interventions focusing on smoking among high-risk subgroups of AANHPI males, given that cancer is the leading cause of death and lung cancer is the leading cause of cancer mortality among Asian American men [26].

We found that experience of discrimination was significantly associated with current smoking among both male and female AANHPI adults, which is consistent with previous studies conducted before and during the COVID-19 pandemic [27-29]. Some AANHPI adults might have adopted smoking as it may have been perceived as a way to cope with increased discrimination experience that they faced during the pandemic [30]. In addition, racial discrimination may also contribute to depressive symptoms by increasing internalized negative self-perceptions and lowering self-esteem [31]. However, elevated levels of depression and anxiety symptoms were significantly associated with higher odds of smoking among AANHPI males only but not among AANHPI females in our study. Current literature shows mixed results in the association between sex, depressive symptoms, and smoking, with some studies reporting a significant smoking-depression association in women but not in men [32-34], some studies indicating a significant association in both sexes [35], and others showing a significant association among males but not in females [36]. These mixed findings could be attributed to the differences in study samples and analysis modeling. Future studies should explore the mechanism for sex differences in the association between smoking and depression.

Regarding cannabis use, we found that Filipino and multicultural groups had the highest rate of cannabis use. The finding of a high rate of cannabis use among Filipinos was consistent with the findings from another study which also found Filipinos had the highest rate of past 12-month drug use (eg, cannabis, cocaine, prescription drugs without physician recommendation, and other drugs) among Asian Americans [19]. The overall prevalence of cannabis use among NHPIs in our study was 5.3%. There are limited studies on cannabis use among NHPIs [37], including one study conducted during the COVID-19 pandemic (between April and November 2021) reporting the rate of lifetime use of illicit substance use (eg, cannabis, opioids) of 35% among NHPIs [18]. Given the small sample of the NHPI group and the underrepresentation of NHPI males in our study, future studies should re-estimate the prevalence of cannabis among NHPIs.

Previous studies that do not take into account ethnic subgroups often reported lower rates of drug use (including cannabis) among female Asian Americans than their male counterparts and females of other race or ethnic groups [19,37]. However, female participants of Asian Indian and Vietnamese groups even seemed to have a higher rate of cannabis use than their male counterparts in our study, indicating the need for taking into account both ethnic subgroups and sex differences in cannabis use among AANHPI adults. We found that younger age (ie, 18‐29 y) and elevated levels of depression and anxiety symptoms were associated with a higher likelihood of cannabis use in both male and female AANHPI adults, which was also consistent with the previous study [19]. However, in our study, discrimination experience and English proficiency were not associated with cannabis among AANHPI males or females, and being born in the United States was only associated with cannabis use among female AANHPI adults.

Similar to smoking and cannabis use, national data (ie, National Survey on Drug Use and Health) often indicate that Asian Americans have the lowest estimated current alcohol use compared to other racial groups (eg, 40.3% among Asian Americans vs 57% among Whites, 45.9% among African Americans, and 49.2% among Hispanics) [38]. Although the overall prevalence of alcohol use among AANHPI adults in this study (37.7%) was comparable to the rate reported from the national data, our study showed that some subgroups (eg, Filipinos or Koreans) had the current alcohol use rate as high as other racial groups. Previous studies also indicate important correlates of alcohol drinking are racial discrimination experience, depression and anxiety symptoms, and nativity [30,39]. This study added more insights to the variation of these correlates by sex, such as a significant association between alcohol use and discrimination experience among AANHPI males but not among AANHPI females.

### Limitations

This study has several limitations. First, despite a large nationally recruited sample of AANHPI adults, the generalizability of the findings to all AANHPI adults across the United States was limited due to a nonprobability sampling approach. In addition, we were unable to estimate the prevalence of substance use among males of some cultural subgroups (ie, Japanese, Filipino, and NHPI) due to a small sample of these groups. Data from this study were collected during the COVID-19 pandemic, so the estimated prevalence of substance use for participants could be lower than when measured at a normal time. Finally, self-reported data are subject to recall and social desirability bias.

### Conclusions and Public Health Implications

Our study reconfirmed the need to address the diversity of AANHPI adults in public health programs, including programs that address substance use and health-related disparities to address various needs across cultural subgroups and general AANHPI adults who experience mental health issues and discrimination. A high rate of substance use among some subgroups of the AANHPI adults has contributed to the disparity in their health outcomes, including lung and liver cancers. Also, differences in cultural norms, stigma, and language barriers could prevent AANHPI adults from accessing social support and treatment services [40]. Yet, AANHPI populations remained underrepresented in clinical research, including the development of substance use prevention and treatment programs [12,41]. Thus, it is essential to develop and implement culturally appropriate prevention and cessation programs to address the needs of AANHPI adults. These programs should engage communities in their development to ensure that intervention content, languages, and delivery methods are aligned with the cultural beliefs, values, norms, and preferences of specific AANHPI groups [41].

## Abbreviations

AANHPI: Asian American, Native Hawaiian, and Pacific Islander
AOR: adjusted odds ratio
COMPASS: COVID-19 Effects on the Mental and Physical Health of Asian Americans & Pacific Islanders Survey Study
NHPI: Native Hawaiian and Pacific Islander
PHQ-4: Patient Health Questionnaire 4
